# Profiling measures of muscle strength and power throughout a 156 km ultra-trail running event

**DOI:** 10.3389/fspor.2025.1734785

**Published:** 2026-01-12

**Authors:** Adrian Markov, Ben J. Edwards, Arnaud Goutorbe, Sonia Gilli, Marcel Lemire, Anh Phong Nguyen, Benoit Mauvieux

**Affiliations:** 1 Normandie Université, UNICAEN, Caen, France; 2 Research Institute for Sport and Exercise Sciences, John Moores University (Liverpool), Liverpool, United Kingdom; 3 MotionLab, Le Mont-sur-Lausanne, Suisse; 4 Faculty of Medicine, Translational Medicine Federation (FMTS), University of Strasbourg, Strasbourg, France; 5 Faculty of Sport Sciences, University of Strasbourg, Strasbourg, France; 6 Institut de Recherche Expérimentale et Clinique, Neuromusculoskeletal Lab, Université Catholique de Louvain, Brussels, Belgium; 7 The Running Clinic, lac Beauport, Quebec, Canada

**Keywords:** fatigue, finisher, non-finisher, trail-running, ultra-endurance

## Abstract

**Purpose:**

Ultra-endurance performance involves complex neuromuscular demands, yet continuous in-race assessment of strength and power development is lacking. This study examined the first-ever continuous profile of neuromuscular fatigue throughout an entire ultra-trail race to understand fatigue mechanisms and inform training and pacing strategies.

**Methods:**

Fifty-five participants (43 men, 12 women; 45.2 ± 13.6 years) attempted six identical 26 km laps with 1,000 m elevation gain and loss per lap, 14 did not complete the course. Maximum knee-extensor and handgrip strength, peak-power output, and jump-height were measured pre-race, after each lap, and 12 h post-race using standardized protocols and linear mixed models.

**Results:**

Knee-extensor strength decreased by ∼41% from pre-race to finish (*p* < .001), with substantial recovery (*Δ*26%–27%) at 12 h post-race. Handgrip strength showed minimal overall decline (*Δ*∼2%–5%), suggesting fatigue localized to the lower limbs. Peak-power and jump-height declined gradually (*Δ*6%–7% from early laps; *p* < .001). Critically, no significant relationship existed between the magnitude of strength loss and final ranking (early and late finishers showed no differences in strength profiles). However, participants who withdrew at lap 5 displayed substantially lower baseline strength (*Δ*27%; *p* = .004) and progressive strength declines compared to finishers, suggesting baseline neuromuscular capacity may influence completion likelihood.

**Conclusion:**

Continuous in-race profiling reveals that ultra-trail running induces substantial and predominantly peripheral neuromuscular fatigue in the lower limbs, with limited systemic effects. While strength loss magnitude does not predict race placement among finishers, lower baseline strength may increase non-completion risk. These findings underscore the importance of targeted strength training and metabolite-clearance strategies (e.g., glycogen replenishment, hydration, recovery) in ultra-endurance preparation.

## 1 Introduction

Ultra-endurance events particularly ultra-trail running have experienced a rise in popularity over the past decade, attracting both elite and recreational runners. With increasing participation attributed to women and master runners notably in ultramarathon races (1, 2). This growing interest reflects the appeal of these extreme endurance challenges, which are typically defined as races exceeding the marathon distance of 42.2 km, or for events with a race duration >6 h, marked by extreme physical, mental and environmental challenges often lasting from several hours to multiple days. Despite this expanding participation, scientific evidence investigating the associated physiological responses and adaptations remains sparse (3). Multidisciplinary performance models (4) have emphasized that ultra-trail performance is complex and dependent on many factors (e.g., muscle force, maximum rate of oxygen consumption, pacing, running technique and nutrition). Within the limited literature investigating ultra-trail running, neuromuscular fatigue has emerged as a critical component warranting further investigation to better understand its impact during such demanding endurance events (5).

The loss of muscle strength and power during prolonged endurance exercise is a complex process and a result of neuromuscular fatigue involving both peripheral and central mechanisms (6, 7). Peripheral fatigue refers to the reduction in force-generating capacity of the muscle fibers themselves, often attributed to metabolic changes, impaired excitation-contraction coupling and structural damage to muscle fibers. Central fatigue, in contrast, involves alterations in the central nervous system activity that lead to a decrease in neural drive to the muscles, resulting in reduced voluntary activation (8–10). Both peripheral and central fatigue contribute to performance decrements in endurance events, but their relative contributions vary depending on several factors such as the environment, psychological factors, perceived exertion, type, duration and intensity of the exercise (11–17). For example, Black and colleagues (13) demonstrated that metabolic and neuromuscular determinants differ systematically across exercise intensities. In trail running specifically, Pastor et al. found that elite trail runners exhibit higher maximal torque and power than road runners (18), and that performance predictors shift with race distance (19). Accordingly, shorter races (<60 km) are predicted by aerobic capacity, whereas longer events (∼100 km) are more influenced by baseline strength and body composition. Recent biomechanical work has further characterized how running kinematics adapt across the intensity spectrum. Accordingly, Monteiro et al. (20) documented systematic changes in stride timing and joint angles with increasing intensity, while Silva et al. (21) reviewed how fatigue modulates ground reaction forces in long-distance runners. Yet despite this understanding of intensity/duration-dependent neuromuscular and biomechanical responses, no study has continuously tracked muscle strength and power throughout an entire ultra-trail event under authentic competition conditions. This represents a critical knowledge gap, as understanding the magnitude and timing of neuromuscular fatigue development during actual racing is essential for preventing dropout, optimizing training, and developing effective race strategies (14, 22–27). The unique six-lap, 156 km design of the present study captures repeated in-race assessments under authentic competition conditions, providing unprecedented insights into fatigue development that distinguishes this work from existing literature.

Building on this novel design, the primary aim of the present study was to map changes in maximal voluntary upper and lower-limb strength (i.e., knee extensors and handgrip) and lower-limb muscle power in a diverse cohort of male and female participants. Based on previous evidence of distinct central and peripheral fatigue processes, we hypothesized that lower-limb strength and lower-limb muscle power would decline progressively with increasing race duration, whereas upper-limb strength would remain stable throughout the event, reflecting differential contributions of fatigue mechanisms to task-specific muscle groups. In this context, we aimed to explore potential strength differences between the dominant and non-dominant leg, hypothesizing that any asymmetric demands of trail running could result in greater strength decline in the dominant leg. The secondary aim was to investigate whether muscle strength predicts race performance, hypothesizing that greater baseline maximal strength and smaller declines in strength from pre- to post-race would be associated with better performance (i.e., faster finishing times). Furthermore, we proposed that a minimum baseline strength and a maximum allowable strength decrement might be necessary to successfully finish an ultra-trail race, with runners presenting lower baseline strength and larger strength losses at higher risk of poor performance or non-completion.

## 2 Methods

### 2.1 Participants

Fifty-five participants of the competitors (43 men: mean ± SD values for age 45.6 ± 14.6 years, height 1.76 ± 0.1 m, weight 70.3 ± 7.8 kg, BMI 22.7 ± 2.0 and mean body fat 9.7% ± 5.4% and 12 women: age 43.8 ± 9.7 years, weight 53.5 ± 5.5 kg, BMI 19.7 ± 1.1 and body fat 17.7% ± 4.8%) participated in this study (Table 1). Fourteen out of 55 participants did not complete the entire race and dropped out due to different reasons (muscle/systemic fatigue, gastrointestinal symptoms). This study is part of a multidisciplinary assessment protocol, performed during the first “Trail Scientifique de Clecy in 2021” with only muscle performance values presented here. A detailed listing of all inclusion and exclusion criteria is listed elsewhere (5). Inclusion criteria: participants were healthy (see exclusion criteria), aged 25-70 years old, could read and speak the French language, were experienced ultra-trail runners who had completed at least two ultra-trail races [including one over 100 miles [>160 km] and one over 62 miles [<160 km] prior to the study], but had not complete any ultra-endurance events within two months of this ultra-trail race. They had registered with the intention to complete the full 156 km race distance, ensuring focus on performance over the entire competition. Exclusion criteria: Suffered any recent injuries or infections before the race. Any cardiac or extracardiac contraindications to intense physical activity (e.g., pregnancy, significant inflammatory, renal, cardiac, neurological disease) as well as current medical treatment. Prior to participation, written informed consent was obtained from all participants. The experimental procedure was approved by the *Comité de Protection des Personnes Ouest III* (21.09.61/SIRIPH 2G21.01586.000009) and conducted in accordance with the latest Declaration of Helsinki.

**Table 1:** The characteristics of participants.

Characteristics	Value
Number of participants (*n*)	55
Age (years)	45.2 ± 13.6
Body mass (kg)	55.0 ± 10.1
Body mass index (kg/m^2^)	22.0 ± 2.1
Male	43
Female	12
Finisher	41
Total mean ± SD race finishing times (h:min:s)	25:29:48 ± 04:33:58
Total race duration male winner (h:min:s)	17:49:14
Total race duration female winner (h:min:s)	20:50:49
First Finisher (h:min:s)	17:49:14
Last Finisher (h:min:s)	35:55:21
Non-finisher (completed 2 laps)	1
Non-finisher (completed 3 laps)	3
Non-finisher (completed 4 laps)	6
Non-finisher (completed 5 laps)	4

Data are presented as mean ± standard deviation.

### 2.2 Procedure

**Figure 1:**
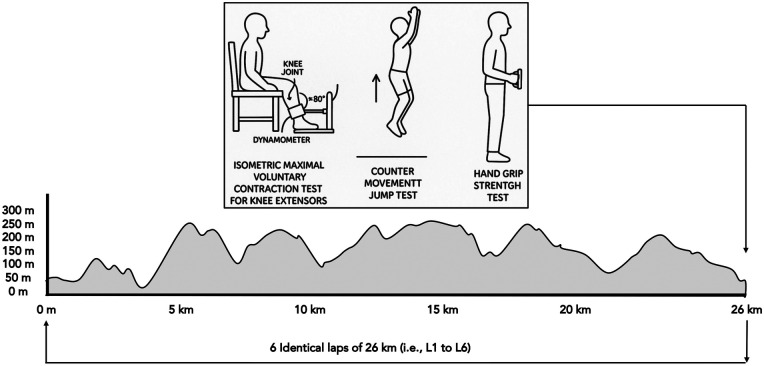
Schematic overview of the protocol and route: a 26 km loop with 1,000 m of ascent and decent to be completed 6 times. Measurements (dominant and non-dominant leg strength, handgrip strength and jump power) were taken before, during (6 points, after each lap) and after the race (12-h after the finish). Full details of the protocol are available in open access (5).

The race started on November 11, 2021, at 14:30 h and covered a challenging 156 km course with 6,000 m of elevation gain and loss. It was structured as six identical 26 km laps, each featuring 1,000 m of elevation gain and loss. For standardization, none of the participants were allowed to use trekking poles during the entire race. For a schematic overview of the protocol see Figure 1. This single-group study utilized a repeated measures design, with data collection occurring at up to eight time points (pre-race, after each completed lap [L1–L6] and 12 h post-race). Body mass was measured in kilograms using the BC545N (Tanita) scale. Body composition was assessed on the morning of the race and at the finish line using an mBCA 525 (Seca) impedance meter in the supine position to determine the proportion and distribution of fat, water and muscle. It is a non-invasive technique validated against the gold standard (27). Due to logistical constraints at the race finish line, including participant fatigue and time limitations immediately following race completion, handgrip strength, countermovement jump height and peak power measurements could not be consistently obtained from all participants 12 h post-race. Therefore, these variables are not reported for the final measurement occasion, while isometric leg strength was prioritized and successfully completed for all finishers. Based on varying individual performances and the distinction between finishers and non-finishers (DNF), the number of measurements differed among participants. Runners were required to be self-sufficient between refreshment points, with a water supply available midway through each lap. At the end of each lap, participants had access to a standard race refreshment station offering beverages, food and personal items. Following refueling, runners proceeded to a designated scientific zone where the race clock was paused for testing. Upon completion of the scientific assessments, participants embarked on their next lap. In preparation for the event, participants were advised to adhere to their regular training regimens and follow their customary pre-race nutrition strategies in the weeks leading up to the race.

### 2.3 Muscle strength and muscle power measurements

Isometric maximal voluntary contraction of the knee extensors was assessed for both dominant and non-dominant leg. Participants were seated in a quadriceps chair in a standardised position with their arms crossed, hands on their shoulders, back against the backrest and knee bent at 90°. A dynamometer [Takei® TK200, Takei Scientific Instruments, Japan; measurement range 5–100 kg-force (kgf) in 0.1 kgf increments] mounted on the armrest of the quadriceps chair was used to record the maximum force produced (expressed in kgf). All participants performed two alternating maximal 5–7 s (s) contractions per leg to ensure a plateau in peak force for accurate measurement (28), with the best result used for analysis. Handgrip strength was assessed using a grip dynamometer (Grip, K-Invent, Montpellier, France) with participants seated, arms at 90° elbow flexion and instructed to squeeze maximally for 5 s (29), recording the maximal force produced in kgf. For lower limb muscle power assessment, participants performed three squat jumps on a portable force plate system (Vald, FD4000, Brisbane, Australia; unit: Watts). Participants placed their hands on their hips and were instructed to jump as high as possible during each repetition, with no real-time feedback on jump height. Vertical ground reaction force (GRF) and center of pressure (COP) data were recorded at 1,000 Hz using dual uniaxial force plates (ForceDecks FD4000) connected to iOS and Windows software for data acquisition. The system calculates velocity by integrating acceleration derived from force and body mass, enabling calculation of peak power as the product of force and velocity during the concentric jump phase. A standardized protocol with 30 s rest intervals between trials and 1–2 min of rest between tests was followed to ensure data reliability and validity (29–31). The highest peak power value from the three trials was used for statistical analysis.

### 2.4 Statistical analyses

Data were analysed using the Statistical Package for Social Science (IBM SPSS, Chicago, IL, USA, version 29.0). To account for the varying number of completed laps among participants, a linear mixed model (LMM) approach was employed. The LMM included fixed effects for measurement interval (i.e., pre-race, L1-L6 and 12 h post-race: we will refer to this as “time” in the manuscript) and random effects for participants. Separate models were run for isometric maximal voluntary contraction of the knee extensors, handgrip strength and lower limb muscle power (i.e., squat jump). An autoregressive covariance structure (AR1) were used to model the correlation between repeated measurements, as it assumes stronger correlations between temporally closer measurements (32). This structure is particularly suitable for longitudinal data with repeated measures over time. For the model estimation, Restricted Maximum Likelihood (REML) with a maximum of 300 iterations were used to ensure convergence and robust parameter estimates (33). Residual normality was assessed using the Shapiro–Wilk test (*p* = .014), which indicated a statistically significant deviation. However, visual inspection of Q-Q plots revealed only minor deviations at the distribution tails, with most residuals aligning closely with the diagonal line. Homogeneity of variances across “time” were tested via Levene's test (*p* = .001), indicating significant heterogeneity. The AR1 structure were retained despite these deviations, because it is robust for estimating fixed effects in larger samples and our primary focus was on identifying temporal trends in muscle strength and power. In the presence of significant main effects for lap, Bonferroni pairwise comparisons were made (34).

Further, regression analyses were conducted to investigate whether the final race placement/ranking is associated with the initial muscular strength of the dominant leg measured before the race or the magnitude of muscle strength decline for each lap (i.e., pre-race to [L1–L6] and post-race [following 12 h of rest and/or sleep]). Only the dominant leg was used to avoid collinearity issues arising from the high correlation between bilateral strength measures, ensuring clarity and interpretability of the results (35). Additionally, a repeated measure two-way ANOVA was performed to compare the first half of the finishers with the second half of the finishers, the first 10 with last 10, as well as the first 5 with the last 5 finishers. This was conducted to examine whether the degree of muscle force development differs throughout the race, between those who finish earlier vs. later.

To allow for a more nuanced understanding of how muscle strength changes differ among participants with varying levels of race completion, while at the same time accounting for the unequal group sizes a more descriptive approach was used (36). Accordingly, participants were grouped based on the number of completed laps (six laps vs. one, two, three, four and five laps). For each group, we calculated mean values and standard deviations of maximum muscle strength of the dominant leg at each measurement point. Pairwise comparison and percentage changes were computed between consecutive measurements and from pre- to 12 h post-race for each group. To compare between groups, we focused on the common laps completed by all groups being compared. For instance, when comparing the six-lap group with the three-lap group, only the first three laps were considered. This approach avoids the survivor bias that would arise from pooling DNF across different withdrawal points and ensures that all compared participants have identical exposure to race stressors up to each measurement. Additionally, we calculated effect sizes (Cohen's d) for the changes in muscle strength between groups and interpreted as small (<.50), medium (<.80), or large (≥.80) (37). Statistical significance was set at *p* < .05.

## 3 Results

**Figure 2:**
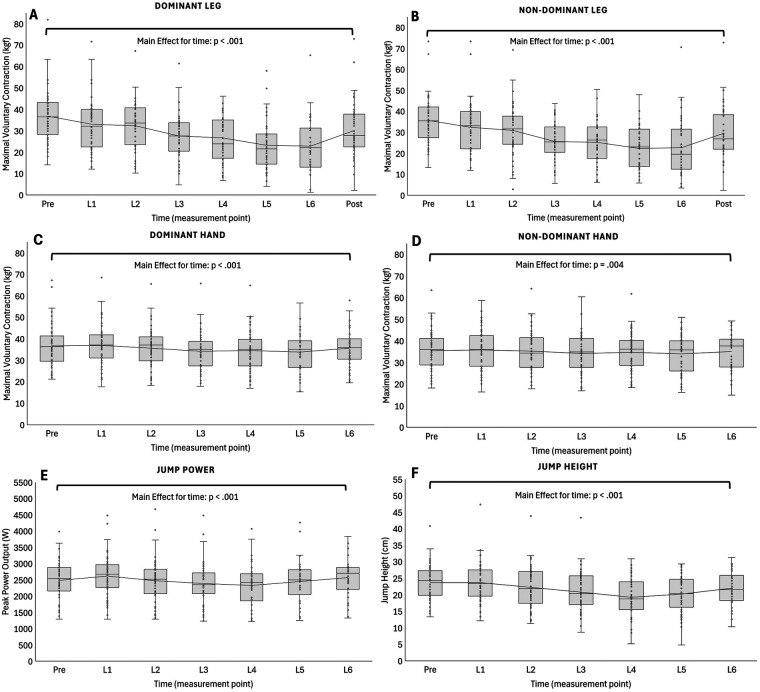
Longitudinal changes in muscle strength and power. **(A,B)** Maximal voluntary contraction of the knee extensors (dominant and non-dominant legs), measured from pre-race to 12 h post-race. **(C,D)** Handgrip strength (dominant and non-dominant hands), measured from pre-race to lap six (race finish). **(E,F)** Peak power output and jump height, assessed from pre-race to lap six. Data are presented as boxplots with individual values overlaid. All variables showed a main effect of time (*p* < .005), reflecting progressive declines, with no differences observed between dominant and non-dominant limbs. L denotes lap; L1–L6 represent measurements obtained after each completed lap.

**Figure 3:**
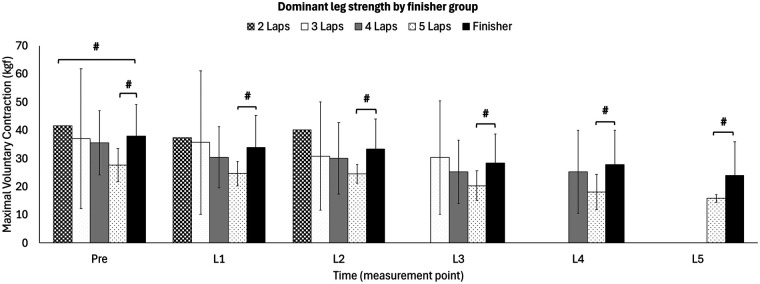
Longitudinal changes in maximal voluntary contraction of the knee extensors (dominant leg) from pre-race to completion points (L1–L5) in 2- to 5-lap finishers, compared with those that finished the race (indicated with blacked out rectangle). #: *p* < .005 vs. overall finishers at the corresponding time point.

For more detailed information on estimated marginal means and 95% confidence interval, please refer to Table 2 as well as Figures 2, 3 for a graphical illustration of the main results. Both absolute and relative values were used for statistical analyses. However, for reasons of practical interpretability, we present the results based on absolute values as the findings did not differ substantially between approaches. For interested readers, all data and results including those based on relative values are openly available via the Open Science Framework.

**Table 2:** Estimated marginal means and 95% confidence interval (CI) for all measures and intervals from pre-race to L6; and knee-extensors strength also at 12 h after finishing. L denotes lap; L1–L6 represent measurements obtained after each completed lap.

Intervals	Isometric maximal voluntary contraction of the knee extensors						Hand grip strength						Counter movement jump					
	Dominant leg (kgf)			Non-dominant leg (kgf)			Dominant hand (kgf)			Non-dominant hand (kgf)			Peak power output (W)			Jump height (cm)		
	Mean	95% CI		Mean	95% CI		Mean	95% CI		Mean	95% CI		Mean	95% CI		Mean	95% CI	
		Lower	Upper		Lower	Upper		Lower	Upper		Lower	Upper		Lower	Upper		Lower	Upper
PRE	37.0	33.7	40.3	35.8	32.7	38.8	36.7	34.2	39.2	35.6	33.2	38.1	2,480.3	2,308.2	2,652.4	23.8	22.2	25.4
L1	33.0	29.8	36.3	32.4	29.3	35.4	37.0	34.5	39.5	36.0	33.5	38.4	2,619.0	2,446.9	2,791.1	23.7	22.1	25.3
L2	32.0	28.7	35.3	30.9	27.9	34.0	35.7	33.2	38.2	35.2	32.7	37.7	2,473.7	2,301.6	2,645.8	22.2	20.6	23.9
L3	27.1	23.8	30.4	25.0	21.9	28.1	34.3	31.8	36.8	34.2	31.7	36.7	2,379.4	2,207.0	2,551.8	20.7	19.1	22.4
L4	26.9	23.5	30.2	24.9	21.8	28.1	34.3	31.8	36.8	34.7	32.2	37.1	2,349.8	2,176.3	2,523.3	19.5	17.9	21.1
L5	23.1	19.7	26.5	21.9	18.7	25.2	34.0	31.5	36.5	34.5	32.0	37.0	2,423.0	2,248.0	2,598.1	19.6	18.0	21.3
L6	22.0	18.5	25.4	21.3	17.8	24.7	34.8	32.3	37.4	34.9	32.4	37.4	2,525.2	2,347.6	2,702.7	21.4	19.7	23.1
12-h POST	29.8	26.5	33.2	29.7	26.6	32.8												

### 3.1 Time-dependent changes in muscle strength and power

There was a main effect for time for isometric maximal voluntary contraction of the knee extensors (dominant leg: F_7, 210_ = 24.883, *p* < .001; non-dominant leg: F_7, 168_ = 15.095, *p* < .001 respectively) handgrip strength (dominant hand: F_6, 166_ = 7.823, *p* < .001; non-dominant hand: F_6, 179_ = 3.344, *p* = .004, respectively) and lower limb muscle power (peak power output: F_6, 156_ = 12.826, *p* < .001; jump-height: F_6, 161_ = 15.739, *p* < .001 respectively). From pre to L6 muscle strength declined, this was irrespective of leg-side (*Δ*41%, *p* < .001 respectively). Dominant leg strength declined pre-race to L1 (*Δ*11%, *p* < .001), from L2 to L3 (*Δ*12%, *p* < .001) and L4 to L5 (*Δ*9%, *p* < .001), while non-dominant leg strength only decreased from L2 to L3 (*Δ*11%, *p* < .001). From L6 to 12 h post-race, knee extensors strength increased by *Δ*26%–29% relative to L6 values (*p* < .001), irrespective of leg-side. Handgrip strength varied throughout the race, with values intermittently rising above and dropping below baseline, but a decline from pre-race to L6 was observed only for the dominant hand (5%, *p* = .029). Also, dominant handgrip strength declined from L1 to L3 and from L2 to L3 (*Δ*7%, *p* < .001 and *Δ*4%, *p* = .020 respectively). Meanwhile, non-dominant handgrip strength declined from pre-race to L3 (*Δ*4%, *p* = .037), L1 to L3 (*Δ*5%, *p* = .002) and L1 to L5 (*Δ*4%, *p* = .040) but went back towards baseline from L5 to L6. Regarding muscle power, there was a gradual decline in peak power output and jump-height from L1 to L4 with differences from L1 to L2 (*Δ*6%, *p* < .001 and *Δ*6%, *p* = .030 respectively) and L2 to L3 (*Δ*4%, *p* = .014 and *Δ*7%, *p* = .002 respectively).

### 3.2 Finisher vs. non-finisher isometric maximal voluntary contraction of the dominant knee extensors

A pairwise comparison of isometric maximal voluntary contraction of the dominant knee extensors showed a difference between the finisher group (*n* = 41) and the 2-lap-DNF at pre-race (41.60 vs. 37.99 kg, respectively; *Δ*9%, *p* = .045; ES = .46). With respect to the 5-lap-DNF there was a difference in isometric maximal voluntary contraction of the dominant knee extensors compared to finishers at all timepoints of measurement: pre-race (27.68 vs. 37.99 kg; *Δ*27%, *p* = .004; ES = 1.15), L1 (24.68 vs. 33.92 kg; *Δ*27%, *p* = .002; ES = 1.07), L2 (24.55 vs. 33.32 kg; *Δ*26%, *p* = .001; ES = 1.04), L3 (20.40 vs. 28.43 kg; *Δ*28%, *p* = .013; ES = .98), L4 (18.10 vs. 27.88 kg; *Δ*35%, *p* = .011; ES = 1.01) and L5 (15.85 vs. 23.93 kg; *Δ*34%, *p* < .001; ES = .94). There was no difference in isometric maximal voluntary contraction of the dominant knee extensors between finishers and the 3- or 4-lap-DNF for either of the timepoints of measurement.

### 3.3 Early vs. late finishers

A two-way ANOVA revealed no difference in isometric maximal voluntary contraction of the dominant leg between the first half of the finishers and the second half of the finishers, between the first 10 and last 10, as well as between the first 5 and last 5 finishers before or during the race. Also, the magnitude of strength loss from pre-race to L2 explained only a small proportion of the variance in the final ranking (*R*^2^ = 0.11, *p* > .05), which is only marginally higher than other comparisons (all *R*^2^ < 0.10, *p* > .05) and should be interpreted with caution.

## 4 Discussion

The main aim of this study was to map out the magnitude of muscle force development throughout an entire ultra-trail running race. Therefore, we have assessed maximal voluntary muscle strength of the dominant and non-dominant leg and hands as well as measures of muscle power at multiple time points throughout a 156 km race (six 26 km laps). We hypothesized that knee extensor strength and muscle power would decline with increasing race duration, while handgrip strength would remain stable, reflecting different contributions and profiles of peripheral and central fatigue across muscle groups. Our results showed significant changes across progressive laps in knee extensor strength, handgrip strength and lower limb muscle power. Additionally, muscle strength differed between participants who completed 5 laps and those who finished all laps at all timepoints. However, there was generally no meaningful association between muscular strength loss and final race placement.

### 4.1 Main effects

#### 4.1.1 Muscle strength

With respect to maximum knee extensor strength, there was a continuous decrease across all measurements [pre-race to finish (L6)], irrespective of the leg-side, with isometric maximal voluntary contraction of the knee extensors significantly decreasing by approximately 41% of its baseline capacity. However, from finish (L6) to 12 h post-race there was evidence of recovery, with significantly higher muscle strength values post-race compared to L6 (*Δ*27%–29%). This pattern is in line with the existing literature (38–40) and seems to be reasonable considering the high muscular demands of ultra-endurance running where the legs naturally bear most of the mechanical and metabolic stress. Accordingly, the continuous mechanical stress, concentric and eccentric muscle contractions as well as the high metabolic demands placed on the leg muscles during trail-running are very likely to induce an accumulation of metabolites but also energy depletion and structural damage to the muscle fibers (14, 41–44). Another interesting finding is the parallel decline and subsequent recovery in both dominant and non-dominant legs. Given the asymmetric demands of trail running terrain, we hypothesized that trail runners might rely more on their dominant leg when navigating uneven surfaces, crossing small obstacles, or managing up- and downhill running (45). As such, the dominant leg might be preferentially used for pushing off on uphill sections, stabilizing on descents or for stepping over roots and rocks which, in consequence, could have led to a more pronounced strength decline in the dominant leg. However, our results suggest that the loss of muscle strength develops symmetrically and leg-independently, thus affecting both limbs to a similar extent. This is in agreement with Mohr et al. (46) who compared kinematic adaptations to running on stable vs. unstable surfaces in male and female runners. The authors reported that running on an unstable, irregular, and compliant surface resulted in a more crouched gait pattern, characterized by increased leg flexion and forward trunk lean, as well as higher stride-to-stride variability in most principal running movements. Accordingly, our results may be explained by an adopted gait pattern and well-developed bilateral coordination among participants, which may serve as a robust strategy to counteract surface-induced disturbances.

While there was a main effect for time, handgrip strength showed a markedly different pattern compared to leg strength (Figure 2). Throughout the race, there was a gradual decline from L1 to L3 (*Δ*5%–7%), no marked difference from L3 to L5 and a slight increase back towards baseline from L5 to L6. Thus, the actual overall decline from pre-race to L6 in handgrip strength was only marginal (*Δ*∼2%–5%), irrespective of hand-side. This would suggest that in runners who were not using poles in the event, neuromuscular fatigue was predominantly localized to the lower limbs, with limited systemic effects on upper body musculature. However, our results highlight the importance of central fatigue mechanisms taking place during ultra-trial events. Thus, it could be argued that the observed reduction in muscle strength of the lower- but not upper limbs indicate a domination of peripheral fatigue mechanisms. However, this would be contrary to the findings of others. Martin et al. investigated neuromuscular fatigue during a 24 h treadmill run in experienced male ultramarathon runners, reported a significant reduction in maximal voluntary contractions of the knee extensors and plantar flexors as well as a significant reduction in voluntary activation and electromyography activity. They found limited evidence of peripheral fatigue, notably no low-frequency fatigue and only moderate reduction in maximal force production ability. Based on these results, the authors suggested central fatigue as the predominant factor of neuromuscular fatigue following an ultra-endurance task (39). This would be in line with studies by others (47). However, a direct comparison of the current study with others is challenging, as the preservation of handgrip strength throughout the ultra-trail race does not allow us to conclude a predominance of peripheral fatigue mechanisms. Instead, central and peripheral processes likely interact in a task-dependent manner, and future studies should incorporate direct peripheral fatigue markers (e.g., twitch interpolation or muscle biopsies) to disentangle their relative contributions.

Another explanation for our results could potentially be that the race started at 14:30 h and laps 2 and 3 were completed at night. A hypothesis is that this timing might contribute to circadian influences on neuromuscular performance. Previous research suggests that physical performance and muscle function may be impaired during nocturnal hours, attributed to fluctuations in hormonal levels and central nervous system activity (48, 49) as well as sleep deprivation (50, 51). However, it is important to note that the observed strength decreases should be interpreted within the context of combined physiological stressors. While isolated circadian variations typically account for 4%-13% differences in muscle strength between morning and evening performance (52) sleep deprivation studies report strength decreases ranging from 3% to 17% depending on the type and duration of sleep restriction (50, 52, 53). Our participants experienced the synergistic effects of extreme physical exertion, metabolic depletion and prolonged wakefulness. Therefore, the magnitude of performance decline observed likely exceeds what would be expected from circadian or sleep factors alone, representing the cumulative impact of multiple physiological stressors during ultra-endurance competition. Conversely, measurements taken at the final time point (L6), showed an increase towards baseline in handgrip strength. This phenomenon may result from a motivational surge, as runners approach the finish line. Where their heightened arousal and effort transiently enhance neuromuscular output, despite accumulated fatigue. Such effects have been shown previously, suggesting a link between neuromuscular performance and psychological factors such as motivation (54, 55). Together, these findings underline the value of incorporating multiple intra-race measurement points to capture the complex interplay between physiological fatigue, circadian rhythms and psychological factors influencing muscular performance in ultra-endurance runners.

#### 4.1.2 Muscle power

With respect to muscle power, our results showed a gradual decline in jump height from L1 to L4 (*Δ*18%), paralleled by a gradual decrease in peak power output (*Δ*10%). Surprisingly, our results showed a partial recovery for both jump height and peak power output from L4 to L6, perhaps reflecting a participant's adopted pacing strategy or a shift in energy substrate utilization and improved fatigue resistance with progressing race length. Other studies, investigating the effects of long-duration endurance events on muscle power did not find such results (56, 57). These studies typically report a continuous decline in muscle strength and power throughout the race. However, it is important to note that most studies do not conduct measurements during the event but focus on comparisons before and after the race. Therefore, the continuous measurements taken within our study may provide insights that are not captured in studies with only pre-vs.-post assessments. Another interesting finding was that jump height was stable from pre-race to L1, while peak power output increased (*Δ*6%). This is might due to a post-activation performance enhancement effect, which is characterized by an acute enhancement of muscle performance following a conditioning activity (58). Alternatively, the apparent increase may reflect learning effects between the pre-race and L1 measurements. Regardless of the increase being only evident in peak power output and not in jump height, this finding suggests that muscles with inherent power-generating capacity fail to convert this power into enhanced performance. This in turn, might be due to alterations in motor unit recruitment and synchronization, muscle fiber types, muscle metabolic characteristics and/or movement efficiency under fatigued conditions (59–61).

The distinct patterns between muscle power and maximum muscle strength from L4 to L6 is another interesting finding within this study. Accordingly, maximum muscle strength continued to decline while muscle power recovered within the last third of the race. Based on the literature, we would speculate that this is might be due to changes in muscle-tendon stiffness and/or a reallocation of energy, which might differentially affect sustained force production compared to brief explosive efforts (62, 63). This speculation aligns with recent findings from our study protocol, which included an assessment of longitudinal changes in Achilles tendon and triceps surae muscle architecture (64). Here, we observed significant biphasic changes in Achilles tendon cross-sectional area (AT-CSA), which could have influence on muscle-tendon stiffness. Initially, the authors noted a decrease in AT-CSA, most likely due to mechanical stress, which was followed by an increase that persisted post-race, suggesting adaptive mechanotransduction. While muscle-tendon stiffness was not directly measured, these changes in tendon properties within this ultra-trail race could have altered the mechanical behavior of the muscle-tendon unit. Therefore, the observed changes in AT-CSA could imply alterations in stiffness, which is crucial for optimizing force transmission and running efficiency. Accordingly, the dynamic changes in muscle-tendon architecture observed in our study protocol support the idea that changes in muscle-tendon stiffness and energy resource reallocation could differentially affect sustained force production compared to brief explosive efforts during ultramarathons.

### 4.2 Associations between muscle strength and race outcome

The secondary aim of this study was to examine how muscle strength relates to race performance, specifically focusing on race completion and finishing position. We investigated whether baseline maximal strength and the degree of strength decline from pre- to post-race differed between finishers and non-finishers, and how these factors might be linked to dropout and sustained effort throughout the race (Section 4.2.1). Furthermore, we explored whether muscle strength and its changes across the race predict finishing times among runners who completed the event, comparing early and late finishers to better understand performance nuances (Section 4.2.2). These analyses highlight the complex physiological and strategic factors contributing to ultra-trail running success.

#### 4.2.1 Finisher vs. non-finisher

The observed differences in baseline strength values and fatigue patterns among participants who completed varying numbers of laps could be accounted to multiple factors. Including gender, body mass composition, body mass and race-specific demands. Notably, all runners who withdrew after 5 laps cited generalized exhaustion as their primary reason for dropout, while earlier dropouts reported mixed causes (e.g., mechanical issues like foot pain, digestive problems or vomiting). This highlights the importance of ultra-trail running experience and suggests that a minimum inclusion criterion of two completed races may be insufficient to adequately prepare participants for the demands of a +100 km ultra-trail event. However, the small sample sizes in all the DNF-groups limit the generalizability of these findings. Finishers’ maximum strength of the dominant leg showed a gradual decline from pre-race to L6 (*Δ*41%). A similar pattern was found for those who dropped out after L5. Therefore, it could be argued that both groups applied a well-paced effort throughout the race (Figure 3). However, finishers showed a higher baseline strength measure compared to those who completed 5 laps only (i.e., 37.99 ± 11.16 vs. 27.68 ± 5.91 kg; *Δ*27%, *p* = .004; ES = 1.16). Notably, when normalizing strength values to body mass, this difference between groups was no longer significant (*Δ*15%, *p* > .05; ES = .64). This suggests that body mass and gender composition (three of four 5-lap DNF participants were female) may partly explain the observed baseline disparities. It should be emphasized that while absolute strength differences diminished when accounting for body mass, a lower absolute strength capacity might still entail a mismatch between the athlete's physical capacity and the absolute demands of the race (e.g., overcoming fixed external loads like steep gradients). This substantial fatigue, particularly in absolute terms, could explain the inability to complete the final lap. Participants completing three or four laps showed similar baseline strength measures within the dominant leg compared to the finisher group (37.03 ± 24.83 vs. 35.53 ± 11.39 and 37.99 ± 11.16 kg, respectively). This might indicate that other factors beyond neuromuscular function influenced their race outcomes. Similarly, a recent study which aimed to identify differences between finishers and DNF in a 100 km ultra-endurance race in male ultra-endurance runners observed that finishers had significantly less fluid intake before the day of competition. As well as lower systolic blood pressure, body weight, body mass index and faster half-marathon times prior to the race when compared to DNF. Therefore, the authors stated these findings as predictive factors for a successful completion of ultra-endurance races (65). However, our study did not find any pre-race difference between finisher and DNF in body weight, body mass index, systolic blood pressure or *International Trail Running Association*-performance index, emphasizing that the outcome of ultra-endurance races is influenced by a complex interplay of multiple factors beyond these physiological measures. Accordingly, it seems that successful ultra-trial performance depends on a complex interplay of different factors as for instance pacing strategy, baseline strength capacity and physiological preparation/fatigue resistance (14, 66). Thus, finishers might have found an optimal balance between these factors, allowing them to maintain a steady effort throughout the race. In contrast, those who dropped out prior to the end of the race might have lacked one or more of these factors. For instance, the five-lap-DNF's baseline strength capacity, together with a gradual fatigue throughout the race could indicate that participants within this group have pushed beyond their sustainable limits somewhere within or during the race.

#### 4.2.2 Early vs. late finisher

The findings of this study highlight the complexity of predicting race performance in ultra-trail events. Regression analyses revealed that there was generally no meaningful association between muscular strength loss and final race placement. The largest effect observed was a weak relationship between the magnitude of strength loss from pre-race to measurement point 3 (L2) and the final ranking (*R*^2^ = 0.111). While numerically slightly above 0.1, this difference from values below 0.1 is minimal and should not be overinterpreted. It indicates only a small proportion of explained variance. One possible explanation for this weak trend is that runners who experience more pronounced strength loss in the early stages of the race may be less able to sustain optimal performance, but this remains speculative. Overall, our data suggest that muscular fatigue does not substantially determine final ranking, with all other comparisons yielding similarly low R² values. This supports our suggestion that factors beyond baseline physical capacities and/or the magnitude of strength loss during the race likely play a greater role in performance. In line with this, a two-way ANOVA comparing the first half, the first 5 and 10 finishers with the second half, last 5 and 10 finishers, respectively, showed no significant differences in the development of muscle strength throughout the race. This absence of significant main effects or interactions indicates that the degree of strength loss is relatively similar regardless of finishing position, underlining that other factors, such as pacing and/or recovery strategies, may have a greater impact on race outcomes (67). In this context, although we measured muscle strength and power again at 12 h follow-up, we did not examine whether the degree of recovery (*Δ*performance from finish to recovery) predicts race outcomes. Given the varied, self-selected recovery strategies and the exploratory design of these assessments, any association could be confounded by unstandardized post-race practices. Therefore, we recommend that future research systematically control and record recovery modalities to evaluate their predictive value for race performance.

### 4.3 Limitations

We exclusively used isometric knee extensor strength measurements. Previous research suggests that declines in isometric and dynamic muscle strength after trail running can differ in magnitude, with isometric tests potentially overestimating strength loss compared to dynamic assessments. As a result, our findings may not fully represent the range of muscle strength changes occurring during the race (68). Secondly, the partly exploratory nature of our analyses, particularly the group comparisons between finishers and non-finishers with unequal sample sizes, poses significant statistical challenges. These comparisons should be interpreted with caution, as participants’ characteristics (e.g., age, sex, training/racing experience) differed between groups, and the LMM assumptions of residual normality (Shapiro–Wilk, *p* = .014) and variance homogeneity (Levene's test, *p* = .001) were formally violated. Although the covariance structure (AR1) and REML estimation are robust for fixed effects in larger samples, the heterogeneity of variances and residual non-normality may affect the precision of estimates. While our participants ranged from 45.2 ± 13.6 years of age and included both men and women, the small subgroup sizes precluded meaningful statistical analyses of age and sex effects. The observed gender distribution reflects the typical participation patterns in ultra-trail events, where male participants comprise approximately 70%–80% of the field (2). Thirdly, the longitudinal design with repeated measurements during an ultra-trail race inherently introduces variability (e.g., pacing strategies, environmental factors), which could not be fully controlled. Hence, another potential limitation of the present study is the lack of systematic data collection on participants’ food, fluid and caffeine intake strategies during the race. Although standardized refreshment stations were available after each lap and runners were self-sufficient between these points, individual variability in nutritional intake may have affected strength and power test outcomes. Importantly, this variability reflects real-race conditions where runners self-manage their nutrition based on individual preferences and needs, thereby making our results representative of actual ultra-trail racing. However, as highlighted in a publication by our team conducted on this protocol (44), which aimed to continuously monitor blood glucose levels, no increased risk of hypo- or hyperglycemia was observed during the running phases of the exercise (excluding stops for scientific measurements and refueling) compared to resting values. Lastly, the exclusion of trekking poles, while important for standardizing conditions, may reduce the applicability of our findings to ultra-trail events where pole use is prevalent, as poles can significantly modify biomechanical loading and fatigue distribution during prolonged mountainous running. These limitations highlight the need for further research with larger, more balanced samples and stricter control of confounding variables. Nevertheless, the highly significant main effects (*p* = .001) and the robustness of LMMs to moderate assumption violations support the validity of our primary findings regarding temporal declines in muscle performance

### 4.4 Practical implications

The gradual decline in leg strength throughout the race highlights the importance of strategies to manage neuromuscular fatigue. In this context, brief periods of recovery (active and/or passive) with a focus on strategies that address both peripheral and central fatigue mechanisms (e.g., metabolite clearance, glycogen replenishment, hydration or muscle damage repair, rest and sleep) seem to be crucial. With respect to preparation and training, runners and coaches should be aware that implementing regular strength training into the weekly training routine could substantially enhance running performance. In this sense, recent literature highlighted that 2–3 training sessions per week, including low to high intensity resistance training as well as plyometric exercises significantly improve running economy in ultra-endurance runners (69–72). Further, since muscle strength declines symmetrically in both legs during ultra-trail running, coaches should prioritize balanced strength training protocols for both limbs to prevent asymmetric fatigue patterns and associated injury risk. Moreover, pacing strategies should be carefully developed and evaluated during training in alignment with each runner's individual neuromuscular strength profile to optimize performance and delay fatigue.

## 5 Conclusions

This study is the first to map muscle force development continuously throughout an entire ultra-trail race. Our results reveal a pronounced decline in lower limb muscle strength and power indicating complex interactions between peripheral and central neuromuscular fatigue mechanisms, with limited impact on upper body musculature. Importantly, the findings show that neuromuscular fatigue alone does not strongly predict race outcome, implicating additional physiological and strategic factors in ultra-endurance performance. These findings have practical relevance for designing targeted training, pacing, and recovery interventions aimed at enhancing performance and reducing injury risk in ultra-endurance runners. Further investigation focusing on variables such as age, training status, pre-race physiological markers, and the application of complementary neuromuscular assessments could provide deeper insight into determinants of race success.

## Data Availability

The datasets supporting this study are openly available on the Open Science Framework at https://osf.io/v9eug/overview.

## References

[1] Scheer V. Participation trends of ultra endurance events. Sports Med Arthrosc. (2019) 27(1):3–7. 10.1097/JSA.0000000000000198 30601393

[2] Hoffman MD Fogard K. Demographic characteristics of 161-km ultramarathon runners. Res Sports Med. (2012) 20(1):59–69. 10.1080/15438627.2012.634707 22242737

[3] Vancini RL Dos Santos Andrade M Barbosa de Lira CA Nikolaidis PT Knechtle B. Is it possible to age healthy by performing ultra-endurance exercises? Int J Sport Stud Health. (2022) 4(1):e122900. 10.5812/intjssh.122900

[4] Balducci P Clemencon M Trama R Blache Y Hautier C. Performance factors in a mountain ultramarathon. Int J Sports Med. (2017) 38:819–26. 10.1055/s-0043-112342 28799161

[5] Mauvieux B Hingrand C Drigny J Hodzic A Baron P Hurdiel R Study of the kinetics of the determinants of performance during a mountain ultramarathon: multidisciplinary protocol of the first trail scientifique de clécy 2021. JMIR Res Protoc. (2022) 11(6):E38027. 10.2196/38027 35704381 PMC9244647

[6] Behrens M Gube M Chaabene H Prieske O Zenon A Broscheid K-C Fatigue and human performance: an updated framework. Sports Med. (2023) 53(1):7–31. 10.1007/s40279-022-01748-2 36258141 PMC9807493

[7] Burnley M Jones A. Power–duration relationship: physiology, fatigue, and the limits of human performance. Eur J Sport Sci. (2018) 18:1–12. 10.1080/17461391.2016.1249524 27806677

[8] Boyas S Guével A. Neuromuscular fatigue in healthy muscle: underlying factors and adaptation mechanisms. Ann Phys Rehabil Med. (2011) 54(2):88–108. 10.1016/j.rehab.2011.01.001 21376692

[9] Lambert E Gibson A Noakes T. Complex systems model of fatigue: integrative homoeostatic control of peripheral physiological systems during exercise in humans. Br J Sports Med. (2004) 39:52–62. 10.1136/bjsm.2003.011247 PMC1725023 15618343

[10] Tornero-Aguilera JF, Jimenez-Morcillo J, Rubio-Zarapuz A, Clemente-Suarez V. Central and peripheral fatigue in physical exercise explained: a narrative review. Int J Environ Res Public Health. (2022) 19(7):3909. 10.3390/ijerph19073909 35409591 PMC8997532

[11] Alix-Fages C Jimenez-Martinez P Souza de Oliveira D Möck S Balsalobre-Fernandez C Del Vecchio A. Mental fatigue impairs physical performance but not the neural drive to the muscle: a preliminary analysis. Eur J Appl Physiol. (2023) 123(8):1671–84. 10.1007/s00421-023-05189-1 36988671

[12] Pageaux B Lepers R. Fatigue induced by physical and mental exertion increases perception of effort and impairs subsequent endurance performance. Front Physiol. (2016) 7:587. 10.3389/fphys.2016.00587 27965592 PMC5126404

[13] Black MI Jones AM Blackwell JR Bailey SJ Wylie LJ McDonagh STJ Muscle metabolic and neuromuscular determinants of fatigue during cycling in different exercise intensity domains. J Appl Physiol. (2017) 122(3):446–59. 10.1152/japplphysiol.00942.2016 28008101 PMC5429469

[14] Millet GY. Can neuromuscular fatigue explain running strategies and performance in ultra-marathons? The flush model. Sports Med. (2011) 41:489–506. 10.2165/11588760-000000000-00000 21615190

[15] Lloyd A Raccuglia M Hodder S Havenith G. Interaction between environmental temperature and hypoxia on central and peripheral fatigue during high-intensity dynamic knee extension. J Appl Physiol. (2016) 120(6):567–79. 10.1152/japplphysiol.00876.2015 26769955

[16] Vøllestad N. Metabolic correlates of fatigue from different types of exercise in man. Fatigue. (1995) 384:185–94. 10.1007/978-1-4899-1016-5_15 8585450

[17] Temesi J Besson T Parent A Singh B Martin V Brownstein CG Effect of race distance on performance fatigability in male trail and ultra-trail runners. Scand J Med Sci Sports. (2021) 31(9):1809–21. 10.1111/sms.14004 34170574

[18] Pastor FS Besson T Berthet M Varesco G Kennouche D Dandrieux P-E Elite road vs. Trail runners: comparing economy, biomechanics, strength, and power. J Strength Cond Res. (2022) 37:181–6. 10.1519/JSC.0000000000004226 36515604

[19] Pastor FS Besson T Varesco G Parent A Fanget M Koral J Performance determinants in trail-running races of different distances. Int J Sports Physiol Perform. (2022) 17(6):844–51. 10.1123/ijspp.2021-0362 35213820

[20] Monteiro AS Galano JP Cardoso F Buzzachera CF Vilas-Boas JP Fernandes RJ. Kinematical and physiological responses of overground running gait pattern at different intensities. Sensors (Basel). (2024) 24(23):7526. 10.3390/s24237526 39686063 PMC11644620

[21] Silva M Afonso J Ramirez-Campillo R Fernandes RJ Conceicao F. Effects of fatigue on ground reaction forces-related variables: a systematic review in long-distance runners: una revisión sistemática en corredores de fondo. Eur J Hum Mov. (2024) 53:5-31. 10.21134/eurjhm.2024.53.8

[22] McLean S Fellin RE Suedekum N Calabrese G Passerallo A Joy S. Impact of fatigue on gender-based high-risk landing strategies. Med Sci Sports Exercise. (2007) 39(3):502–14. 10.1249/mss.0b013e3180d47f0 17473777

[23] Moran K Marshall B. Effect of fatigue on tibial impact accelerations and knee kinematics in drop jumps. Med Sci Sports Exercise. (2006) 38(10):1836–42. 10.1249/01.mss.0000229567.09661.20 17019307

[24] Costa R Hoffman M Stellingwerff T. Considerations for ultra-endurance activities: part 1- nutrition. Res Sports Med. (2018) 27:166–81. 10.1080/15438627.2018.1502188 30056753

[25] Pérez A Ramos-Campo DJ Freitas TT Rubio-Arias JA Marin-Cascales E Alcaraz PE. Effect of two different intensity distribution training programmes on aerobic and body composition variables in ultra-endurance runners. Eur J Sport Sci. (2018) 19:636–44. 10.1080/17461391.2018.1539124 30381001

[26] Zaryski C Smith D. Training principles and issues for ultra-endurance. Curr Sports Med Rep. (2005) 4:165–70. 10.1097/01.CSMR.0000306201.49315.73 15907270

[27] Hoffman MD Lebus DK Ganong AC Casazza GG Van Loan M. Body composition of 161-km ultramarathoners. Int J Sports Med. (2010) 31(2):106–9. 10.1055/s-0029-1241863 20222002

[28] Harnie J Cattagni T Cornu C McNair P Jubeau M. Acute effect of tendon vibration applied during isometric contraction at two knee angles on maximal knee extension force production. PLoS One. (2020) 15(11):e0242324. 10.1371/journal.pone.0242324 33186411 PMC7665630

[29] Kamimura T Ikuta Y. Evaluation of grip strength with a sustained maximal isometric contraction for 6 and 10 s. J Rehabil Med. (2001) 33(5):225–9. 10.1080/165019701750419626 11585154

[30] Collings TJ Lima YL Dutaillis B Bourne MN. Concurrent validity and test–retest reliability of VALD ForceDecks’ strength, balance, and movement assessment tests. J Sci Med Sport. (2024) 27(8):572–80. 10.1016/j.jsams.2024.04.014 38777737

[31] Robertson CM Pullinger SA Robinsom WR Smith ME Burniston JG Edwards BJ. Circadian variation in muscle force output in males using isokinetic, isometric dynamometry: can we observe this in multi-joint movements using the muscleLab force-velocity encoder and are they similar in peak and magnitude? Chronobiol Int. (2024) 41(5):709–24. 10.1080/07420528.2024.2348011 38722075

[32] Littell R Pendergast J Natarajan R. Modelling covariance structure in the analysis of repeated measures data. Stat Med. (2000) 19(13):1793–819. 10.1002/1097-0258(20000715)19:13<1793::AID-SIM482>3.0.CO;2-Q 10861779

[33] Kenward M Roger J. Small sample inference for fixed effects from restricted maximum likelihood. Biometrics. (1997) 53(3):983–97. 10.2307/2533558 9333350

[34] Midway S Robertson M Flinn S Kaller M. Comparing multiple comparisons: practical guidance for choosing the best multiple comparisons test. PeerJ. (2020) 8:e10387. 10.7717/peerj.10387 33335808 PMC7720730

[35] Dormann CF Elith J Bacher S Buchmann C Carl G Carre G Collinearity: a review of methods to deal with it and a simulation study evaluating their performance. Ecography. (2013) 36(1):27–46. 10.1111/j.1600-0587.2012.07348.x

[36] Murphy K. In praise of table 1: the importance of making better use of descriptive statistics. Ind Organ Psychol. (2021) 14:461–77. 10.1017/iop.2021.90

[37] Cohen J. The Effect Size. Statistical Power Analysis for the Behavioral Sciences. Abingdon: Routledge (1988). p. 77–83.

[38] Millet GY Lepers R. Alterations of neuromuscular function after prolonged running, cycling and skiing exercises. Sports Med. (2004) 34:105–16. 10.2165/00007256-200434020-00004 14965189

[39] Martin V Kerherve H Messonnier LA Banfi J-C Geyssant A Bonnefoy R Central and peripheral contributions to neuromuscular fatigue induced by a 24-h treadmill run. J Appl Physiol. (2010) 108(5):1224–33. 10.1152/japplphysiol.01202.2009 20167672

[40] Millet GY Lepers R Maffiuletti NA Babault N Martin V Lattier G. Alterations of neuromuscular function after an ultramarathon. J Appl Physiol. (2002) 92(2):486–92. 10.1152/japplphysiol.00122.2001 11796655

[41] Garbisu-Hualde A Santos-Concejero J. What are the limiting factors during an ultra-marathon? A systematic review of the scientific literature. J Hum Kinet. (2020) 72:129–39. 10.2478/hukin-2019-0102 32269654 PMC7126261

[42] Millet G Tomazin K Verges S Vincent C Bonnefoy R Boisson R-C Neuromuscular consequences of an extreme mountain ultra-marathon. PLoS One. (2011) 6(2):e17059. 10.1371/journal.pone.0017059 PMC3043077 21364944

[43] Saugy J Place N Millet GY Degache F Schena F Millet GP. Alterations of neuromuscular function after the world’s most challenging mountain ultra-marathon. PLoS One. (2013) 8(6):e65596 10.1371/journal.pone.0065596 PMC3694082 23840345

[44] Parent C Mauvieux B Lespagnol E Hingrand C Vauthier JC Noirez P Glycaemic effects of a 156-km ultra-trail race in athletes: an observational field study. Sports Med. (2024) 54(8):2169–84. 10.1007/s40279-024-02013-4 38555307

[45] Dhawale N Venkadesan M. How human runners regulate footsteps on uneven terrain. eLife. (2021) 12:e67177. 10.7554/elife.67177 PMC10030110 36810138

[46] Mohr M Peer L De Michiel A van Andel S Federolf P. Whole-body kinematic adaptations to running on an unstable, irregular, and compliant surface. Sports Biomech. (2023) 24(10):1–15. 10.1080/14763141.2023.2222022 37317805

[47] Temesi J Rupp T Martin V Arnal PJ Feasson L Verges S Central fatigue assessed by transcranial magnetic stimulation in ultratrail running. Med Sci Sports Exerc. (2014) 46(6):1166–75. 10.1249/MSS.0000000000000207 24195865

[48] Atkinson G Reilly T. Circadian variation in sports performance. Sports Med. (1996) 21:292–312. 10.2165/00007256-199621040-00005 8726347

[49] Edwards B Jim W and Reilly T. The effects of circadian rhythmicity and time-awake on a simple motor task. Chronobiol Int. (2007) 24(6):1109–24. 10.1080/07420520701795316 18075802

[50] Craven J McCartney D Desbrow B Sabapathy S Bellinger P Roberts L Effects of acute sleep loss on physical performance: a systematic and meta-analytical review. Sports Med. (2022) 52:2669–90. 10.1007/s40279-022-01706-y 35708888 PMC9584849

[51] Fullagar H Skorski S Duffield R Hammes D Coutts AJ Meyer T. Sleep and athletic performance: the effects of sleep loss on exercise performance, and physiological and cognitive responses to exercise. Sports Med. (2015) 45:161–86. 10.1007/s40279-014-0260-0 25315456

[52] Douglas CM Hesketh SJ Esser KA. Time of day and muscle strength: a circadian output? Physiology (Bethesda). (2021) 36(1):44–51. 10.1152/physiol.00030.2020 33325817 PMC8425416

[53] Easow J Bommasamudram T Munnilari M Adhikari R Edwards BJ Raghurama Nayak K Implications of sleep loss or sleep deprivation on muscle strength: a systematic review. Sleep Breath. (2025) 29(4):242. 10.1007/s11325-025-03413-0 40663194 PMC12263768

[54] Matta P-M Glories D Alamia A Baures R Duclay J. Mind over muscle? Time manipulation improves physical performance by slowing down the neuromuscular fatigue accumulation. Psychophysiology. (2023) 61(4):e14487. 10.1111/psyp.14487 38015102

[55] Morton RH. Deception by manipulating the clock calibration influences cycle ergometer endurance time in males. J Sci Med Sport. (2009) 12(2):332–7. 10.1016/j.jsams.2007.11.006 18356107

[56] LucaAnson JG Palmer CD Hellemans IJ Cotter JD. The impact of 100 h of exercise and sleep deprivation on cognitive function and physical capacities. J Sports Sci. (2009) 27:719–28. 10.1080/02640410902798167 19437188

[57] Rousanoglou EN Noutsos K Pappas A Bogdanis G Vagenas G Bayios IA Alterations of vertical jump mechanics after a half-marathon mountain running race. J Sports Sci Med. (2016) 15(2):277–86. 27274665 PMC4879441

[58] Boullosa D. Post-activation performance enhancement strategies in sport: a brief review for practitioners. Hum Mov. (2021) 22(3):101–9. 10.5114/hm.2021.103280

[59] Cormie P McGuigan M Newton R. Developing maximal neuromuscular power. Sports Med. (2011) 41:17–38. 10.2165/11537690-000000000-00000 21142282

[60] Mitchell E Martin NR Bailey SJ Ferguson RA. Critical power is positively related to skeletal muscle capillarity and type I muscle fibers in endurance-trained individuals. J Appl Physiol. (2018) 125(3):737–45. 10.1152/japplphysiol.01126.2017 29878875

[61] Vanhatalo A Black MI DiMenna FJ Blackwell JR Schmidt JF Thompson C The mechanistic bases of the power–time relationship: muscle metabolic responses and relationships to muscle fibre type. J Physiol. (2016) 594(15):4407–23. 10.1113/JP271879 26940850 PMC4967754

[62] Knicker A Renshaw I Oldham AR Cairns SP. Interactive processes link the multiple symptoms of fatigue in sport competition. Sports Med. (2011) 41:307–28. 10.2165/11586070-000000000-00000 21425889

[63] Petersen K Hansen CB Aagaard P Madsen K. Muscle mechanical characteristics in fatigue and recovery from a marathon race in highly trained runners. Eur J Appl Physiol. (2007) 101:385–96. 10.1007/s00421-007-0504-x 17661071

[64] Drigny J Remilly M Hingrand C Mauvieux B. Longitudinal changes in achilles tendon and triceps surae muscle architecture during a 156-km mountain ultramarathon. J Appl Physiol. (2024) 137(5):1182–93. 10.1152/japplphysiol.00347.2024 39052821

[65] Belinchón-deMiguel P Tornero-Aguilera JF Dalamitros AA Nikolaidid PT Rosemann T Knechtle B Multidisciplinary analysis of differences between finisher and non-finisher ultra-endurance mountain athletes. Front Physiol. (2019) 10:1507. 10.3389/fphys.2019.01507 31920712 PMC6914837

[66] Hawley J Myburgh KH Noakes TD Dennis SC. Training techniques to improve fatigue resistance and enhance endurance performance. J Sports Sci. (1997) 15(3):325–33. 10.1080/026404197367335 9232558

[67] DE Waal SJ Jacobs SD Lamberts RP. Pacing analysis and comparison of TOP-10 and NOT TOP-10 ultra trail Cape Town 100-km finishers. J Sports Med Phys Fitness. (2025) 65(2):218–24. 10.23736/S0022-4707.24.16203-2 39526871

[68] Koral J Fanget M Imbert L Besson T Kennouche D Parent A Fatigue measured in dynamic versus isometric modes after trail running races of Various distances. Int J Sports Physiol Perform. (2022) 17(1):67–77. 10.1123/ijspp.2020-0940 34359049

[69] Balsalobre-Fernández C Santos-Concejero J Grivas G. Effects of strength training on running economy in highly trained runners: a systematic review with meta-analysis of controlled trials. J Strength Cond Res. (2016) 30:2361. 10.1519/JSC.0000000000001316 26694507

[70] Giovanelli N Taboga P Rejc E Lazzer S. Effects of strength, explosive and plyometric training on energy cost of running in ultra-endurance athletes. Eur J Sport Sci. (2017) 17:805–13. 10.1080/17461391.2017.1305454 28394719

[71] Blagrove RC Howatson G Hayes PR. Effects of strength training on the physiological determinants of middle- and long-distance running performance: a systematic review. Sports Med. (2018) 48(5):1117–49. 10.1007/s40279-017-0835-7 29249083 PMC5889786

[72] Beattie K Kenny IC Lyons M Carson BP. The effect of strength training on performance in endurance athletes. Sports Med. (2014) 44(6):845–65. 10.1007/s40279-014-0157-y 24532151

